# Supplementary material for: High-Resolution Crystal Structure of Muscle Phosphoglycerate Mutase Provides Insight into Its Nuclear Import and Role
Source: Int J Mol Sci. 2022 Oct 30;23(21):13198. doi: 10.3390/ijms232113198 (PMC9656839; doi:10.3390/ijms232113198)
Supplement: Supplementary file 1 [file ijms-23-13198-s001.zip › Supplementary Figure S1.pdf]

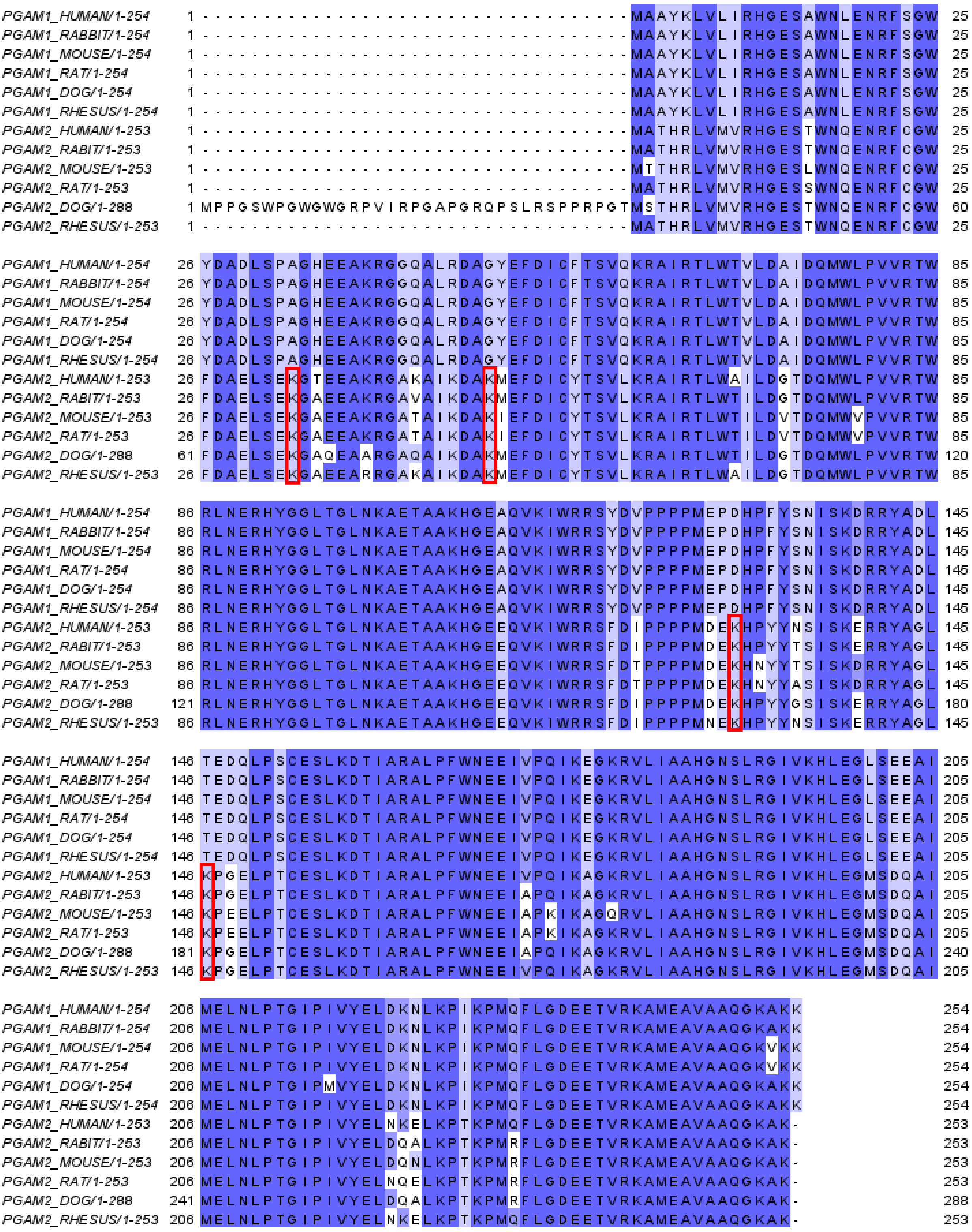

Supplementary Figure S1. Sequence alignment of mammalian PGAM1 and PGAM2. Colored by percentage identity. Lysine residue present in PGAM2 but not PGAM1 highlighted in red.
